# Mechanism of Knee Injuries in the National Basketball Association: A Video-Based Analysis

**DOI:** 10.1155/2024/5594149

**Published:** 2024-04-20

**Authors:** Peyton A. Hull, Andrew P. Collins, Brenden Maag, Jonathan Schwartzman, Zachary A. Gapinski, Benjamin C. Service

**Affiliations:** ^1^Baylor University Medical Center, Department of Orthopaedic Surgery, Dallas, TX, USA; ^2^University of Washington, Department of Orthopaedics and Sports Medicine, Seattle, WA, USA; ^3^University of Central Florida College of Medicine, 6850 Lake Nona Blvd., Orlando, FL, USA; ^4^Orlando Health Jewett Orthopedic Institute, 1222 South Orange Ave. 5th Floor, Orlando, FL, USA

## Abstract

**Background:**

To characterize the mechanism of knee injuries among NBA players during the 2010–2020 seasons using video-based analysis.

**Methods:**

An injury database of NBA players was queried for knee injuries from the 2006-07 to 2020-21 seasons and cross-referenced with NBA injury reports. Youtube.com was searched to identify available injury footage. The mechanism of knee injury during play was analyzed by three independent reviewers. Non-mechanistic data related to the injury was gathered from news reports and official NBA websites.

**Results:**

A total of 2,868 knee injuries occurred in NBA players from 2010 to 2020 seasons; 121 had high quality videos for analysis. The most common mechanism of injury was knee flexion in valgus with internal rotation (27.3%, *p* < 0.001), which was associated with injury to the ACL (55.2%, *p*=0.0001). Injuries occurred most often with control of the ball (62.8%, *p*=0.0064), while on offense (73.6%, *p*=0.0001), and without contact (71.1%, *p*=0.0001). A 28.1% incidence of re-injury was observed in the first 3-year period, and 43.8% of knee injuries required surgery. The average number of games missed due to injury requiring surgery was 55.1 games compared to 8.5 games in those treated nonoperatively (*p* < 0.0001).

**Conclusion:**

Understanding mechanisms of knee injury may guide preventative strategies and injury management programs in NBA players. Video-based analysis reveals the situational characteristics and mechanisms of knee injuries, but further studies are needed to develop injury prevention programs, efficacy of prevention strategies, and rehabilitation to minimize games missed from these injuries.

## 1. Introduction

Basketball continues to grow in popularity and participation amongst all age groups and competition levels [1–3]. The National Basketball Association (NBA) has been instrumental in heightening the popularity of the sport both nationally and worldwide. Unfortunately, the incidence of injuries has increased in the NBA as well. These injuries can contribute to missed game time, financial losses, and the shortening of careers. Knee joint and patellar injuries are less frequent than ankle or lumbar spine injuries but contribute to the greatest number of games missed [4]. All knee injuries represent 18.1% of games missed in the NBA, and 44.1% of game-related injuries are knee injuries. The etiology of knee joint injuries is highly variable. Conditions range from simple bruises to multi-ligamentous tears. Prior studies have demonstrated that younger age and less NBA experience are correlated with earlier return-to-play [5]. Before prevention strategies can be implemented, the mechanism of injuries and risk factors for injury must be identified.

National broadcasting and social media recording have increased the accessibility of injury videos on the Internet. High-quality injury videos may allow medical staff to have greater insight into the mechanism of knee injury allowing optimization of medical care or rehabilitation protocols for these players. Video analysis of injuries in alpine skiing and soccer populations has proven helpful for the mitigation of initial and future injuries [6, 7].

To our knowledge, no studies have used video-based reviews to examine knee injuries in the NBA. We hypothesized that high-quality videographic evidence would be available for most NBA gametime injuries between 2010 and 2020, allowing for an assessment of injury patterns and mechanisms. The primary objective of this study was to characterize NBA in-game knee injuries to identify high-risk situations and common mechanisms of injury. Secondary objectives included the assessment of the number of games missed due to injury, the frequency of surgical intervention, and the economic burden resulting from these injuries. This study will contribute to the literature by providing a video analysis of knee injuries experienced by professional NBA players from the 2010–2020 seasons.

## 2. Methods

The NBA SportsReference injury database was queried for all knee injuries occurring between the 2010-11 and 2019-20 seasons. All knee injuries from this database were cross-referenced with official NBA injury data to ensure validity. Each injury was searched for in the https://YouTube.com video database using the athlete's name and “NBA knee injury.” Non-video-based data was collected from NBA injury reports, news articles, and NBA salary statistics websites.

Videos were analyzed by 3 independent reviewers, each utilizing the mechanism-based knee injury classification system described by Hayes et al. [8]. A preliminary assessment of interobserver reliability for reporting knee injury metrics in this study was found to be 93.4%. Discrepancies were settled with simultaneous video analysis and discussion among reviewers. Multiple factors were evaluated including age, laterality, knee position, contact, player action, team position, surgery requirement, and whether the injury was primary or a reinjury within three years. Additionally, the injured structure, games missed, and lost salary were collected.

Inclusion criteria for the study were (1) participants playing in an NBA game during the injury, (2) injuries occurring between 2010–2020, (3) adequate video quality for analysis, and (4) videos available through the public domain. All injuries occurred during official NBA games and no injuries from training sessions were included.

Injuries excluded from the study were those with (1) ambiguous mechanism, (2) poor quality video evidence, (3) disagreement of mechanism, and (4) knee injury secondary to insult of another anatomic region.

### 2.1. Statistical Analysis

All statistical tests were conducted with IBM SPSS Statistics (v27.0, IBM Corp, Armonk, N.Y., USA). Continuous data was analyzed using Student's *t*-tests while discrete and proportional data were compared using chi-squared analyses. Statistical significance was determined at a confidence level of 95% for all tests. Descriptive statistics were assessed for mean values and standard deviations.

## 3. Results

**Figure 1:**
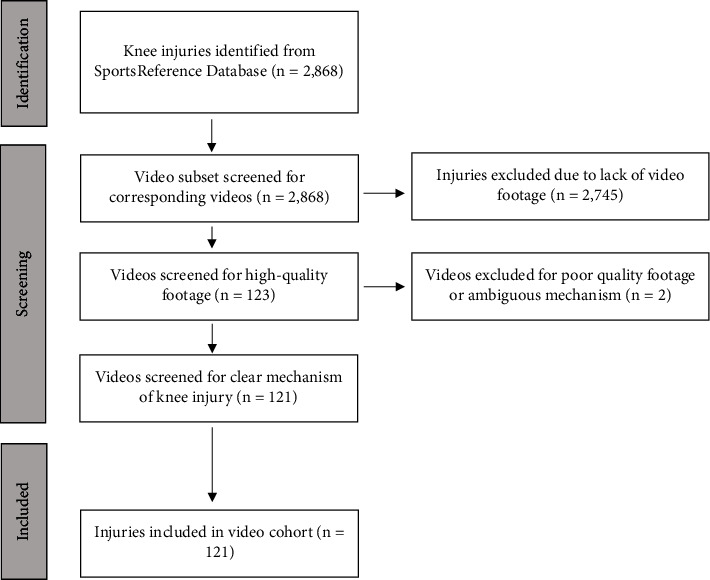
Injury database search results.

A preliminary search yielded 2,868 knee injuries reported to the NBA database during the study period. This search included primary injury, re-injury, and missed play on injured reserve due to surgery or injury recovery. Subsequent filtering of these injuries generated 123 videos. Of these, two videos were excluded from further analysis due to poor video quality and ambiguous injury mechanisms. The data remaining for statistical analysis included 121 videos of 101 unique players with high-quality displays of the mechanism of injury agreed upon by reviewers (Figure 1).

The majority of knee injuries occurred on the left side (55.4%, *p*=0.0174). Injuries occurred most often with control of the ball (62.8%, *p*=0.0064), while on offense (73.6%, *p*=0.0001), and without contact to the ground or from another player (71.1%, *p*=0.0001). The majority of knee injuries did not require surgery (56.2%, *p*=0.0181). Surgical management was required in the other 43.8% of players. Most injuries were primary (69.4%, *p*=0.0001). Re-injury accounted for the other 30.6 percent of the knee injuries with 28.1% of knee injuries observed being re-injured within three years (Table 1).

The most common mechanism of injury occurred with the knee in flexion, valgus, and internal rotation (27.3%, *p*=0.0001). Many injuries did not result in injury to a specific structure and were classified as unspecified bone bruises or ligamentous strains (33.9, *p*=0.0001). The most commonly injured structures with clear pathology were the anterior cruciate ligament (ACL, 24.0%, *p*=0.0001), medial collateral ligament (16.5%, *p*=0.0001), meniscus (10.7%, *p*=0.0001), and the patella (9.1%, *p*=0.0001) (Table 2).

Injuries to the knee flexion in valgus with internal rotation (55.2%, *p*=0.0001) were significantly related to ACL injuries (Table 3).

**Figure 2:**
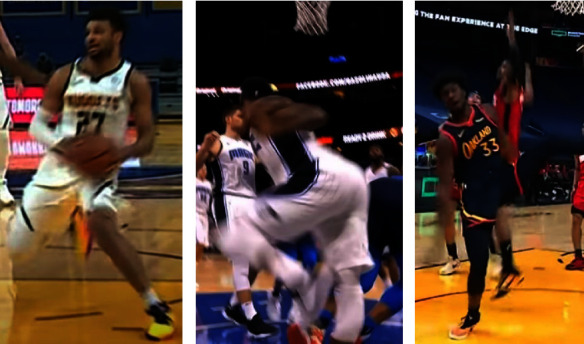
Photos of common mechanisms of injury. (a) Jamal murray ACL injury with knee flexion, valgus, internal rotation. (b) Terrence ross MCL injury with knee hyperextension, valgus. (c) James wiseman meniscus injury with knee flexion, valgus, internal rotation.

Representative injury photos from videos displaying common mechanisms of injury can be seen in Figure 2.

On average, players missed 27.3 games due to knee injuries. Injuries requiring surgical management led to a significantly higher average of games missed at 55.1 versus 8.5 in those managed nonoperatively (*p*=0.0001). Injuries managed operatively were most often primary injuries (67.9%, *p*=0.0134) affecting the ACL and meniscus (47.2% and 17.0%, *p*=0.0001) (Table 4).

Teams on average lost $168K per game missed due to these injuries. The average cost to the teams per knee injury was $3.88M of salary pay (Table 5).

## 4. Discussion

Video analysis of injuries has been used previously to reduce ACL injury among soccer players and alpine skiers [6, 9]. To our knowledge, no studies have used video-based reviews to examine knee injuries in NBA athletes. Knee injuries account for the greatest proportion of missed game time in the NBA [4]. A comprehensive and mechanistic understanding of these insults may benefit athletes and medical staff. This is the first study to present mechanistic injury profiles within this homogenous population, and to our knowledge, the largest video-based injury analysis to date. Over a 15-year study period, high-quality video footage was available for only 4.2% of all knee injuries. The 121 videos analyzed provided a large sample size with robust statistical differences among several outcomes.

Results from this study demonstrated that 71% of injuries did not occur through contact with another player or surface, similar to findings presented by Krosshaug et al. analyzing ACL injuries in basketball players [10]. Furthermore, the ACL was the most commonly identified injured structure. These insults were most likely to occur with knee flexion, internal rotation, and valgus positioning. While significant valgus deformity may be more common in women, movement producing high torque in the frontal and sagittal planes may predispose basketball players to ACL rupture [11–14]. Boden et al. hypothesized that vigorous contraction of the quadriceps on an extended knee may be the main contributor to excessive ACL force based on laboratory studies [15]. Results from our current analysis did not demonstrate this as the most common mechanism of ACL injury in NBA players. Olsen et al. hypothesized that ACL injury could be due to valgus loading with internal or external knee rotation [16]. Ebstrup and Bojsen-Moller proposed notch impingement as a cause of injury [17]. This rotational hypothesis is supported by cadaveric studies placing high stress on the ACL, especially at low flexion angles, similar to the common injury pattern seen in ACL injuries in NBA players [18]. Our results demonstrate these injuries occur more commonly while on offense and in control of the ball. Schultz et al. showed that NBA players with a higher tendency to drive to the basket were more likely to tear their ACL [19]. Post-injury analysis revealed no change in drive tendency following return to sport, despite the high-risk positioning. This information may be used to target players with particular play styles for ACL injury prevention programs.

Drakos et al. demonstrated that from the 1988-89 to 2004-05 NBA seasons, knee and patella injuries accounted for 19.1% of all injuries and 31.7% of games missed. In this epidemiologic study, these injuries resulted in an average of 7.8 games missed per injury [4]. Results from our study demonstrated a similar number of games missed for injuries managed nonoperatively (8.5 games). Based on the data from the current analysis, the average number of games missed for all injuries was 27.3 games. If surgical management was required, the average number of games missed was 55.1 games. This pooled analysis may differ from the Drakos et al. study because their analysis included all injuries reported by athletic trainers [4], which may have included practice or training-related injuries in addition to gameplay injuries.

Our analysis demonstrated that 44% of game-time knee injuries required surgical management. Minhas et al. showed that patients with arthroscopic knee surgery had a significantly greater decline in postoperative performance outcomes at 1- and 3 years following surgery and had shorter career durations following these injuries compared to others [20]. Following knee surgery, quick acceleration and sudden change of motion may contribute to the shear and compressive forces across the tibiofemoral joint. Therefore, the daily pivoting, cutting, and jumping required by the sport may lead to worse postoperative outcomes. Similarly, Busfield et al. analyzed outcomes of ACL reconstruction in NBA athletes, reporting that 22% of players did not return to a sanctioned NBA game, and nearly half experienced a greater than one-point drop in player efficiency rating [21]. As expected, injuries requiring operative management may result in greater games missed and poorer performance upon return to sport.

Graded rehabilitation protocol for return-to-play may increase the likelihood of successful return [22]. Bleakley et al. reported that following an ankle injury, exercise-based rehabilitation may reduce the risk of reinjury [23]. There is no consensus for optimal rehabilitative exercise protocol for knee injury. Furthermore, both medical staff and injured players often feel pressure to prematurely return to sport [24]. Our study showed reinjury events accounted for 30.6% of knee injuries in the NBA and 32% of injuries requiring surgery. The majority of retired NBA players experience residual knee pain and may require surgery after retirement as well, impacting long-term quality of life [25]. Future research reporting details of exercise-based rehabilitation programs following knee injuries may help to form optimal exercise content and training volume consensus to reduce injury-related games missed and reinjury incidence.

Knee and patellar injuries account for over 30% of games missed and often poorer levels of play upon return [4, 21]. These injuries may pose a significant financial burden to teams and players. Unanticipated retirement or copious missed games have financial consequences for players and teams. Despite salaries steadily rising during the last three decades in the NBA, an estimated 60% of former NBA players file for bankruptcy within 5-years of retirement [26]. Analyses from this study showed that game-time knee injuries in the NBA may cost teams an average of nearly $3.88M, which may place stress on their relationship with the player.

We report limitations of this study as well. There was a small yield of knee injuries with high-quality videos over 15 years. Only 4.2% of all recorded injuries had a corresponding publicly available video. This may be due to a lack of attention to minor injuries or those imposed on lesser-known players. This discrepancy could lead to both reporting and selection bias in video-based analyses. The use of public domain videos and exclusion of injuries with poor quality videos may have also led to selection bias. This analysis provides a comprehensive assessment of knee injuries in the NBA, but findings in this homogenous population may not be generalizable to other leagues or the general population.

## 5. Conclusions

This is the first publicly available video-based analysis of the most common mechanisms of knee injury. Improving understanding of knee injury mechanisms during gameplay may provide insight for injury preventative strategies and injury management programs in NBA players. Although video-based analysis does reveal the situational characteristics and mechanisms of knee injuries, further studies are needed to develop injury prevention programs, efficacy of prevention strategies, and rehabilitation to minimize games missed from these injuries.

## Figures and Tables

**Table 1:** NBA knee injury characteristics.

Measure	Outcome	Number (%)	*p* value
Age at time of injury	<20	2 (1.7)	<0.001
	20–24	47 (38.8)	
	25–29	44 (36.4)	
	30–34	24 (19.8)	
	≥35	4 (3.3)	

Laterality	Left	67 (55.4)	0.017
	Right	54 (44.6)	

Position of knee	Flexion	67 (55.4)	0.002
	Extension	48 (40.0)	
	Neutral	6 (5.0)	

Contact	Yes	35 (28.9)	<0.001
	No	86 (71.1)	

Scenario	Landing	33 (27.3)	<0.001
	Jumping	6 (5.0)	
	Cutting	40 (33.1)	
	Direct trauma	42 (34.7)	

Team position	Offense	89 (73.6)	<0.001
	Defense	32 (26.5)	

Player in control of ball	Yes	76 (62.8)	0.006
	No	45 (37.2)	

Surgery required	Yes	53 (43.8)	0.018
	No	68 (56.2)	

Type	Primary injury	84 (69.4)	<0.001
	Reinjury	37 (30.6)	

Did reinjury occur within 3-years?	Yes	34 (28.1)	<0.001
	No	87 (71.9)	

**Table 2:** NBA knee injury mechanism and structure.

Measure	Outcome	Number (%)	*p* value
Mechanism of injury	Knee flexion, varus, external rotation	6 (5.0)	<0.001
	Knee flexion, valgus, internal rotation	33 (27.3)	
	Knee hyperflexion	7 (5.8)	
	Knee hyperextension with valgus	17 (14.0)	
	Knee hyperextension with varus	19 (15.7)	
	Knee hyperextension	14 (11.6)	
	Axial load	8 (6.6)	
	Direct trauma	17 (14.0)	

Injured structure	Patella	11 (9.1)	<0.001
	ACL	29 (24.0)	
	PCL	1 (0.8)	
	MCL	20 (16.5)	
	Meniscus	13 (10.7)	
	Quadriceps tendon	3 (2.5)	
	Popliteus	1 (0.8)	
	Bony fracture	2 (1.7)	
	Non-specific	41 (33.9)	

**Table 3:** Mechanism of distinct injured structures.

Injured structure	Number (% of total injuries)	Mechanism of injury	Number (%)	*p* value
Patellar fracture or dislocation	11 (9.1)	Knee flexion, valgus, internal rotation	4 (36.4)	0.667
		Knee hyperextension with varus	2 (18.2)	
		Knee hyperextension	3 (27.3)	
		Axial load	2 (18.2)	

ACL	29 (24.0)	Knee flexion, varus, external rotation	1 (3.4)	<0.001
		Knee flexion, valgus, internal rotation	16 (55.2)	
		Knee hyperextension with varus	8 (27.6)	
		Knee hyperflexion	2 (6.9)	
		Knee hyperextension	2 (6.9)	

MCL	20 (16.5)	Knee flexion, valgus, internal rotation	5 (25.0)	0.055
		Knee hyperflexion	2 (10.0)	
		Knee hyperextension with valgus	10 (50.0)	
		Direct trauma	3 (15.0)	

Meniscus	13 (10.7)	Knee flexion, varus, external rotation	1 (7.7)	0.872
		Knee flexion, valgus, internal rotation	4 (30.1)	
		Knee hyperextension with valgus	3 (23.1)	
		Knee hyperflexion	1 (7.7)	
		Knee hyperextension	2 (15.4)	
		Direct trauma	2 (15.4)	

**Table 4:** Injuries requiring surgery.

Measure	Outcome	Number (%)	*p* value
Age	—	25.8 ± 4.3 years	—

Games missed	All injuries	27.3 ± 35.1 games	<0.001
	Non-operative management	8.5 ± 12.5 games	
	Surgical management	55.1 ± 39.2 games	

Injury type	Primary	36 (67.9)	0.013
	Reinjury	17 (32.1)	

Injured structure	Patella	5 (9.4)	<0.001
	ACL	25 (47.2)	
	MCL	3 (5.7)	
	Meniscus	9 (17.0)	
	Quadriceps tendon	3 (5.7)	
	Bony fracture	5 (9.4)	
	Non-specific	3 (5.7)	

Mechanism of injury	Knee flexion, varus, external rotation	2 (3.8)	<0.001
	Knee flexion, valgus, internal rotation	18 (34.0)	
	Knee hyperflexion	1 (1.9)	
	Knee hyperextension with valgus	4 (7.5)	
	Knee hyperextension with varus	7 (13.2)	
	Knee hyperextension	6 (11.3)	
	Axial load	4 (7.5)	
	Direct trauma	11 (20.8)	

**Table 5:** NBA knee injury number of games missed and salary data.

Measure	Mean ± standard deviation
Games missed	27.3 ± 35.1
Salary per game	168,196 ± 130,851
Salary lost per injury	3,888,929 ± 6,775,270

## Data Availability

The knee injury data used to support these findings are available from the NBA SportsReference injury database for all knee injuries occurring between the 2010-11 and 2019-20 seasons. Specific data files used to support the findings of this study are available from PH upon request.

## Abbreviations

NBA: National basketball association
ACL: Anterior cruciate ligament.
